# Detection of Tuberculosis Infection Hotspots Using Activity Spaces Based Spatial Approach in an Urban Tokyo, from 2003 to 2011

**DOI:** 10.1371/journal.pone.0138831

**Published:** 2015-09-18

**Authors:** Kiyohiko Izumi, Akihiro Ohkado, Kazuhiro Uchimura, Yoshiro Murase, Yuriko Tatsumi, Aya Kayebeta, Yu Watanabe, Nobukatsu Ishikawa

**Affiliations:** 1 Department of Epidemiology and Clinical Research, the Research Institute of Tuberculosis, Japan Anti-tuberculosis Association (RIT/JATA), Kiyose city, Tokyo, Japan; 2 Nagasaki University Graduate School of Biomedical Sciences, Nagasaki city, Nagasaki, Japan; 3 Department of Mycobacterium Reference and Research, the Research Institute of Tuberculosis, Japan Anti-tuberculosis Association (RIT/JATA), Kiyose city, Tokyo, Japan; 4 Shinjuku City Public Health Center, Shinjuku city, Tokyo, Japan; 5 The Research Institute of Tuberculosis, Japan Anti-tuberculosis Association (RIT/JATA), Kiyose city, Tokyo, Japan; University of Minnesota, UNITED STATES

## Abstract

**Background:**

Identifying ongoing tuberculosis infection sites is crucial for breaking chains of transmission in tuberculosis-prevalent urban areas. Previous studies have pointed out that detection of local accumulation of tuberculosis patients based on their residential addresses may be limited by a lack of matching between residences and tuberculosis infection sites. This study aimed to identify possible tuberculosis hotspots using TB genotype clustering statuses and a concept of “activity space”, a place where patients spend most of their waking hours. We further compared the spatial distribution by different residential statuses and describe urban environmental features of the detected hotspots.

**Methods:**

Culture-positive tuberculosis patients notified to Shinjuku city from 2003 to 2011 were enrolled in this case-based cross-sectional study, and their demographic and clinical information, TB genotype clustering statuses, and activity space were collected. Spatial statistics (Global Moran’s *I* and Getis-Ord Gi* statistics) identified significant hotspots in 152 census tracts, and urban environmental features and tuberculosis patients’ characteristics in these hotspots were assessed.

**Results:**

Of the enrolled 643 culture-positive tuberculosis patients, 416 (64.2%) were general inhabitants, 42 (6.5%) were foreign-born people, and 184 were homeless people (28.6%). The percentage of overall genotype clustering was 43.7%. Genotype-clustered general inhabitants and homeless people formed significant hotspots around a major railway station, whereas the non-clustered general inhabitants formed no hotspots. This suggested the detected hotspots of activity spaces may reflect ongoing tuberculosis transmission sites and were characterized by smaller residential floor size and a higher proportion of non-working households.

**Conclusions:**

Activity space-based spatial analysis suggested possible TB transmission sites around the major railway station and it can assist in further comprehension of TB transmission dynamics in an urban setting in Japan.

## Introduction

Tuberculosis (TB), a bacterial airborne infectious disease caused by *Mycobacterium tuberculosis* (*M*. *tuberculosis*), is one of the world’s deadliest communicable diseases. An estimated nine million people developed TB and 1.5 million died in 2013 worldwide[1]. Although the majority of those TB cases is reported from high incidence countries, TB is still significant public health issue in low and middle incidence countries, including Japan, especially among certain high-risk groups in urban area. Therefore, enhancement of contact investigation targeting those groups is critical to identify infected and infectious TB cases.

Much advance has been made as a result of development of new technologies, such as molecular epidemiological techniques, which have enabled revealing of TB transmission dynamics within social networks [2–5]. Another technique which has recently become available is Geographic Information System (GIS), where geographic data to describe and visualize spatial distributions and to discover spatial association patterns have been applied in TB epidemiological studies. Numerous studies using GIS technology have examined spatial distribution of TB patients [6], socio-economic risk factors [7–9], demographic risk factors [10–13], and TB transmission dynamics in high-incidence areas [14]. In order to assess TB epidemiological situation nationwide, some studies used large data-set such as surveillance data [15] and National Health Insurance data related with TB patients [16]. GIS has also been used in combination with molecular epidemiological techniques for understand the dynamics of TB transmission [17–19].

Most of the studies which have attempted to reveal where transmission was ongoing using spatial analysis were, however, limited, in the sense that spatial information was based on patients’ residential address [17]. TB transmissions occurring outside the households has been indicated [20], but no studies have so far attempted to address this issue. In Japan too, previous studies have also shown that TB transmissions occur in public facilities used by an unspecified number of people, such as saunas, bars, amusement facilities [21] and areas near downtown railroad stations [22].

TB was a top leading cause of death until the 1950s, with a mortality rate of 146.4 per 100,000 population in 1950 in Japan. The rate has since dramatically declined to 1.7 in 2013, as a result of introduction of modern TB control measures and improvements in living standards [23]. Japan is on the track towards becoming a low-burden; in 2013, the notification rate was 16.1 cases per 100,000 population [24]. However, the reduction in the TB mortality and notification rate has stagnated in the recent years, primarily due to the existence of TB high-risk groups, such as elderly population developing active TB from long-standing latent infections [23], homeless people in urban settings [25], and foreign nationals who were born in TB-prevalent countries [24]. Similar to other low and middle TB burden countries, contact investigation and understanding of transmission dynamics have become critical in controlling TB in Japan [26]. TB epidemiological studies using GIS, however, have not been fully conducted in Japan and few of those studies have same limitation as residential based approach.

The objective of our study, therefore, was to identify possible sites of transmission within urban setting using hotspot analysis, in combination with molecular epidemiological and GIS techniques. In order to overcome the limitation of residential based approach, we developed a unique concept of activity space to capture places where TB patients spend most of their waking hours, which may or may not be their residential homes. We further sought to compare spatial distribution patterns among different residential statuses and to describe patient characteristics and urban environmental features of the detected hotspots.

## Methods

This case-based cross-sectional analysis of notified culture-positive TB patients is part of a population-based DNA fingerprinting surveillance of *M*. *tuberculosis* in Shinjuku city, Tokyo, which has been conducted since 2002 [27].

### Setting

**Fig 1:**
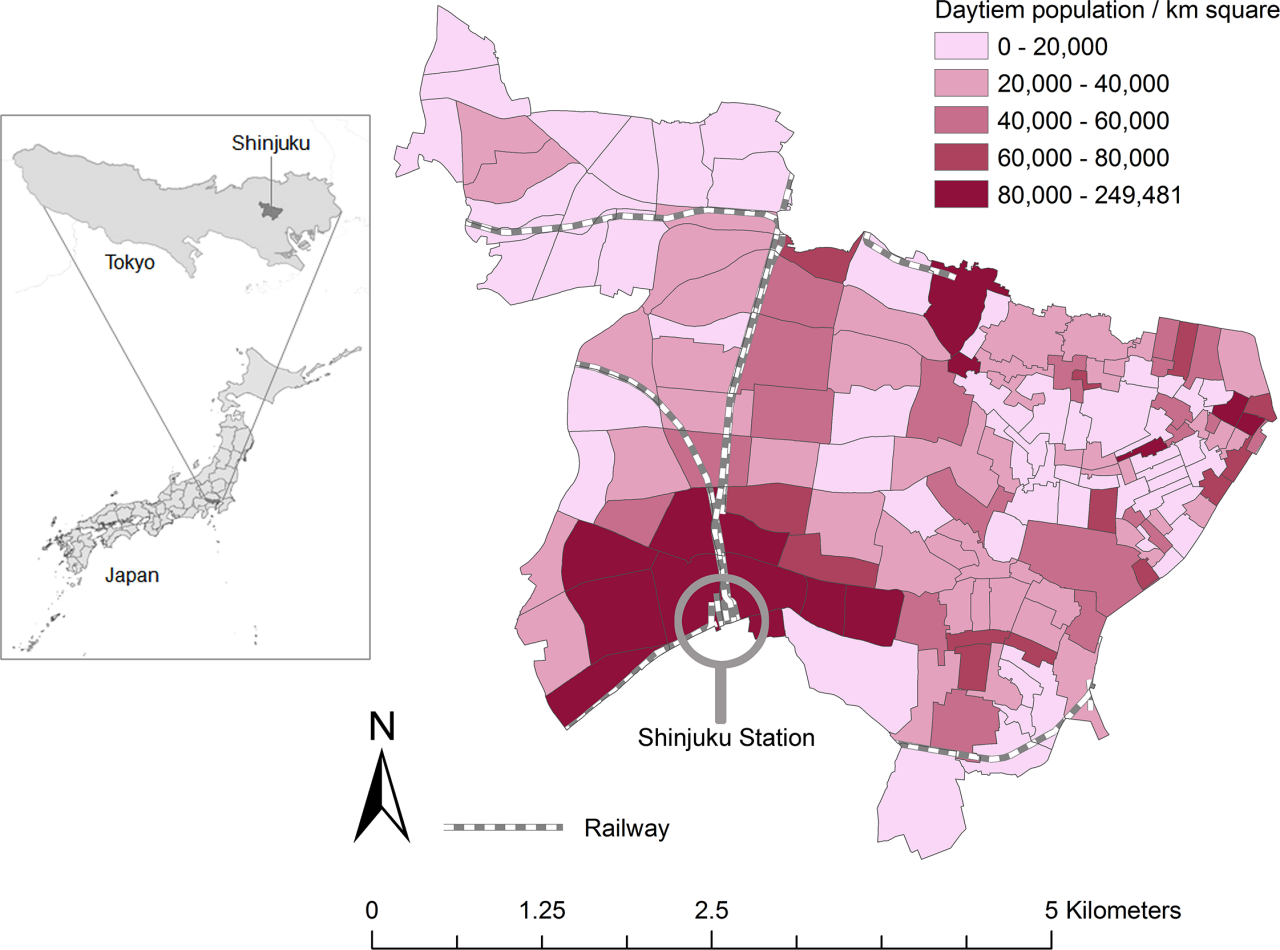
Shinjuku city. Note: Location of Shinjuku city and daytime population density per square kilometer. Note: segments in the city map are the 152 census tracts. Data source: 2005 Japanese Census, the Statistics Bureau, Ministry of Internal Affairs of Japan.

Shinjuku city (18.3 km^2^) is one of the most populous of the cities of Tokyo and, is a home to the Tokyo Metropolitan Government office (Fig 1). A total of 152 census tracts of Shinjuku city consists of diverse urban environments with residential, commercial, and industrial zones. The population of Shinjuku city is 327,712 in 2015, of which 36,016 (11%) are foreign residents [28]. Shinjuku city was ranked fourth among all cities in Japan in terms of high proportion of foreign nationals in 2010 [29]. TB incidence in Shinjuku city was 48.2 per 100,000 population in 2012, which was higher than Tokyo and national average (21.7 and 16.7, respectively).

### Subjects and materials

#### Case data

We included culture-positive TB patients who resided in Shinjuku city and were reported to the Shinjuku PHC between 2003 and 2011 as eligible subjects. Demographic and clinical information, including sex, age, occupation, categories regarding place of birth (born in Japan or foreign born), residential status (domiciled or homeless–in other words, those whose legal address was unknown or unstable in the last two years), and laboratory test results, were collected during the routine patient interview, which is usually conducted within 3 days of diagnosis, by public health nurses at the Shinjuku PHC.

At the same time, additional information regarding “activity space” was collected where a patient was asked to list a maximum of four places where he or she spends most of waking hours in the past two years. The place where the patient spent the longest waking hours was determined as his or her “activity space”, for the purpose of this study. This information was also collected together with the routine interview in order to ensure data validity on activity spaces for estimating possible transmission place to the extent possible.

TB strains isolated from each enrolled patient were subjected to the DNA fingerprinting using IS*6110*-based restriction fragment length polymorphism (IS*6110*-RFLP) analysis [30]. RFLP and spoligotyping were the standard techniques in DNA fingerprinting method in Shinjuku PHC and were available throughout the study period from 2003 to 2011. A genotype cluster was defined as ≥2 isolates, either 1) with ≥6 IS*6110* bands with identical band patterns or 2) with <6 IS*6110* bands with both identical IS*6110* band patterns and spoligotyping patterns. We used criteria of at least 2 isolates identical for ensuring maximum sensitivity for detecting genotype clustering, which is a common practice for TB molecule epidemiological study conducted by Japanese PHCs. All eligible 1,026 culture-positive TB patient in a lump was used for genotype cluster analysis and was classified as genotype clustering or non-clustering. Percentages of genotype clustering were calculated as the number of clustered isolated strains divided by number of all isolated strains from subjects who met the inclusion criteria to the current study.

#### Census tract data

Demographic and urban environmental variables were derived from the 2005 Japanese Census, provided by the Statistics Bureau, Ministry of Internal Affairs of Japan[31]. The 2005 data was chosen as it represented approximately mid-point of the study period. It was used to calculate the variables for each census tract (CT) in Shinjuku city, as shown in Table 1.

**Table 1:** The demographic and urban environmental variables of census tracts.

Variable	Definition
Demographic characteristics	
Daytime population density	Day time population/km² of CT
Density of foreign population	Foreign population/km² of CT
Day and night population ratio	Daytime population/resident population
Percentage of population aged ≥65	Number of persons aged ≥65/resident population ×100
Household density	Number of households/km² of CT
Socioeconomic status	
Total floor area per capita	Total floor area (m²)/number of residents in the household
Percentage of owned house	Number of households with owner-occupants/total number of household ×100/total number of household ×100
Percentage of non-working households	Number of household with only non-working resident/total number of household ×100
Distance to the nearest railway station	Distance of meter from a CT centroid to the nearest railway station

CT = census tract

### Analysis methods

All analyses were based on the CT unit. For each CT, we counted the number of cases who nominated the CT as his or her activity space. We attempted to describe the aggregation and dispersion of TB patients’ activity space and identify their tendency by genotype cluster and residential statuses. Thus the annual TB patient density (the number of TB patients per year, per km^2^) was calculated for each CT. We conducted three steps analysis which were spatial analysis to describe overall spatial aggregation and dispersion, hotspot analysis to detect significant hotspots using spatial statistical method, and assessment of patient characteristics and environmental features of detected hotspots.

#### Spatial analysis

We assessed the level of spatial aggregation of TB patients in the whole study area by examining a spatial autocorrelation using Global Moran’s *I* statistics with row-standardized inverse distance weights matrices [32]. The method measures whether the values of neighboring areas are similar to one another. Thus, significant positive spatial autocorrelation implies that the distribution of TB patients is more spatially aggregation than a random underlying spatial process.

The patient distributions were observed using Inverse Distance Weighted (IDW) interpolation with annual TB patient density. IDW is an interpolation method, and it creates TB patient density surface. In the process, a neighborhood around the interpolated point is identified, and a weighted average is taken of the observation values within this neighborhood. The weight is a function of inverse distance [33].

#### Hotspot analysis

We examined whether patients would form statistically significant local aggregation of activity spaces. CT-level hotspots and coldspots were detected by Getis-Ords G_i_^*^ [34,35], a spatial statistical method available with optimized hotspot analysis function in ArcGIS (Ver.10.2, ESRI Inc., CA, USA). G_i_^*^ statistics is the ratios of the local sum of the values in the vicinity of a distance, in other words the scale of the analysis, to the sum of all values. When the local sum is different than the expected local sum, and that difference is too large to be the result of random chance, a statistically significant Z-score results.

Detected significant hotspots/coldspots imply a high/low value and are surrounded by other features with high/low values. Three confidence interval (CI) levels (90%, 95%, and 99%) are used, with higher confidence levels implying more presumable aggregation of hotspots or coldspots. Detected CTs with more than 90% CI are considered hotspots or coldspots. The scale of analysis was determined through Incremental Spatial Autocorrelation (ISA). This performs the Global Moran’s *I* statistic for a series of increasing distances, measuring intensification of clustering for each distance. The scale of analysis was specified as the distance at which the ISA generated a first Z-score peak, an indicator of pronounced spatial aggregation. The initial distance of ISA was 650 meters, which is a distance at which all features have at least one neighbor. An issue of multiple testing was corrected using False Discovery Rate control, which estimates the expected proportion of incorrectly rejected null hypotheses and adjusts the p-value accordingly [36].

#### Patient characteristics and urban environmental comparison

We compared hotspot analysis result for all patients, patient characteristics within hotspots and outside of hotspots, and demographic and urban environmental variables within hotspots and coldspots. Fisher's exact test and Chi-square test were used for frequency comparison. Unpaired t-test and Wilcoxon-Mann-Whitney test [37] were used for comparing two populations. A p-value of ≤0.05 was considered significant.

The spatial analysis was conducted with ArcGIS and the statistical analysis used STATA (Ver.12.0, StataCorp, TX, USA).

#### Ethics Statement

Clinical, geographic and tuberculosis strain information are collected as part of the routine work of infectious disease control in Shinjuku PHC in accordance with field epidemiological investigation provided in the Infectious Disease Law Article 15, which, in principle, does not require informed consent from the patients. According to the Ethical Guidelines for Epidemiological Research established by Ministry of Education, Culture, Sports, Science and Technology and Ministry of Health, Labour and Welfare, informed consent is also not necessary when using pre-existing data for research purpose. Oral informed consent, however, was obtained from all of the culture-positive TB patients after the PHC staff gave a thorough explanation of study objectives and confidentiality at the interviews. The approvals given by the participants were recorded on the TB patient card. The study protocol including the consent procedure was approved by the Institutional Review Board in Research Institute of Tuberculosis (RIT/IRB 25–26).

## Results

During the study period from 2003 to 2011, 1,026 culture-positive TB patients who resided in Shinjuku city and were reported to the Shinjuku PHC were identified eligible subjects. Among all eligible 1,026 patients, 378 were excluded from present study due to the following reasons: no activity space within Shinjuku city (372 records), and registered multiple times (duplication records) due to suspected TB re-activation (7 records). In the latter case, we included the information of their first TB episode and excluded the following episodes. In the end, 643 records of TB patients were analyzed (Table 2). Comparing all eligible 1,026 patients and 643 enrolled patients, there were no significant differences with regard to sex, age, and residential status. Those who did not meet the criteria were excluded after genotype clustering statuses were classified, in order to examine the spatial distribution of the people who have the attributes that belong to the genotype cluster.

**Table 2:** Demographic and genotyping features of enrolled culture-positive tuberculosis patients registered at the Shinjuku Public Health Center, Tokyo, 2003–2011.

		All enrolled participants								
				General inhabitants		Foreign-born people		Homeless people		Unknown
		n = 643, n (%)		n = 413, n (%)		n = 42, n (%)		n = 184, n (%)		n = 4, n
Sex	Male	491	(76.4)	281	(68.0)	23	(54.8)	184	(100)	3
	Female	141	(21.9)	122	(29.5)	19	(45.2)	0	(0.0)	0
	Unknown	11	(1.7)	10	(2.4)	0	(0.0)	0	(0.0)	1
Age, years	≤19	15	(2.3)	9	(2.2)	4	(9.5)	0	(0.0)	2
	20–39	119	(18.5)	82	(19.9)	23	(54.8)	14	(7.6)	0
	40–59	177	(27.5)	83	(20.1)	12	(28.6)	82	(44.6)	0
	≥60	332	(51.6)	239	(57.9)	3	(7.1)	88	(47.8)	2
Genotype cluster^1^		281	(43.7)	165	(40.0)	7	(16.7)	108	(58.7)	1

^1^ A genotype cluster was defined as ≥2 isolates, either 1) with ≥6 IS*6110* bands with identical band patterns or 2) with <6 IS6110 bands with both identical IS*6110* band patterns and spoligotyping patterns.

In terms of place of birth and residential statuses, all subjects were classified into the following three categories: Japanese-born residents (general inhabitants), foreign-born residents and Japanese-born homeless people–there were no foreign born homeless people in our study and thus the three classifications were mutually exclusive. Of the enrolled 643 culture positive TB patients, 416 (64.2%) were general inhabitants, 42 (6.5%) were foreign-born, 184 were homeless people (28.6%), and four had an unknown residential status. Over the three quarters of patients were males (76.4%); no female homeless patient participated. While number of patients increased with advancing age among general inhabitants and homeless people, 64.3% of foreign-born people were 39 years old or less. In general, 281 (43.7%) of 643 isolates matched DNA band patterns of at least one other patient isolate. Interestingly, the percentage of genotype clustering among foreign born people (16.7%) was far lower than among other residential statuses (40.0% of general inhabitants and 58.7% of homeless people). Activity spaces were generally workplaces for businesspersons, households for retired elderly persons, schools for students, and cardboard city or low-cost lodging for homeless people.

### Spatial analysis

The average annual TB patient density was 3.3 per km^2^. Among all 152 CTs, the median value was 2.3. Global Moran’s *I* analysis for all patients detected spatial autocorrelation with p < 0.01. In other words, the distribution of TB patients in the study area was significantly more spatially clustered than would be expected in a random process (Table 3). Every residential status was spatially auto-correlated to genotype clustering (p-values ranged from <0.01 to 0.04). However, the non-clustered groups of general inhabitants and foreign-born people were not auto-correlated (Table 3).

**Table 3:** Global autocorrelation analysis using Moran’s *I* statistics by residential and genotype cluster statuses.

Residential status	Genotype cluster status	Moran's I	Z-score	*p-*value
All patients		0.308	8.040	<0.01
General inhabitants		0.152	3.667	<0.01
	Clustered	0.083	2.061	0.04
	Non-clustered	0.048	1.305	0.19
Foreign-born people		0.129	3.299	<0.01
	Clustered	0.191	4.731	<0.01
	Non-clustered	-0.021	-0.730	0.47
Homeless people		0.347	9.512	<0.01
	Clustered	0.287	9.122	<0.01
	Non-clustered	0.369	9.385	<0.01

**Fig 2:**
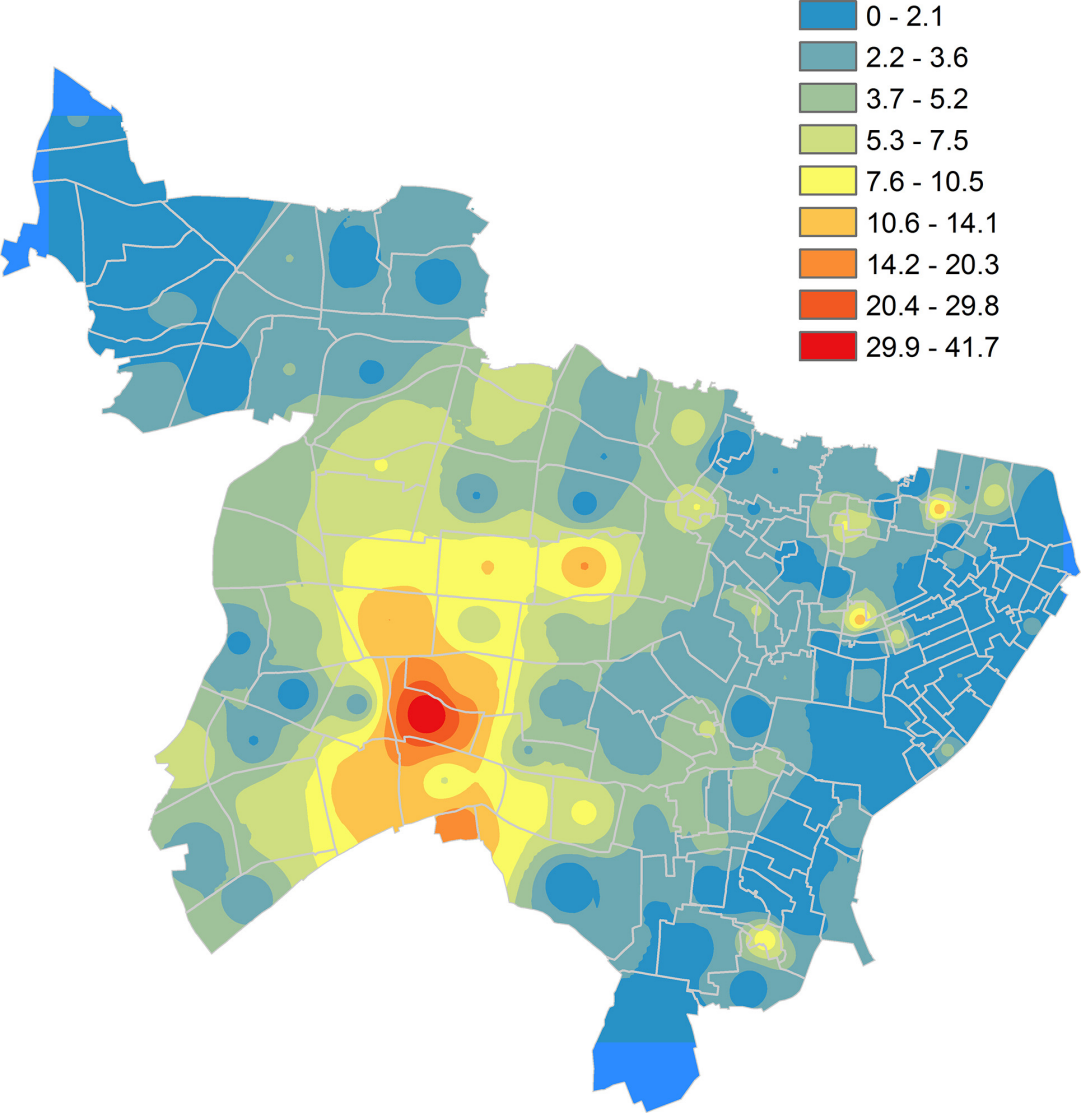
Inverse Distance Weighted interpolated map with annual number of all tuberculosis patients per square kilometer. Note: the color scale indicates the annual number of tuberculosis patients per square kilometer.

**Fig 3:**
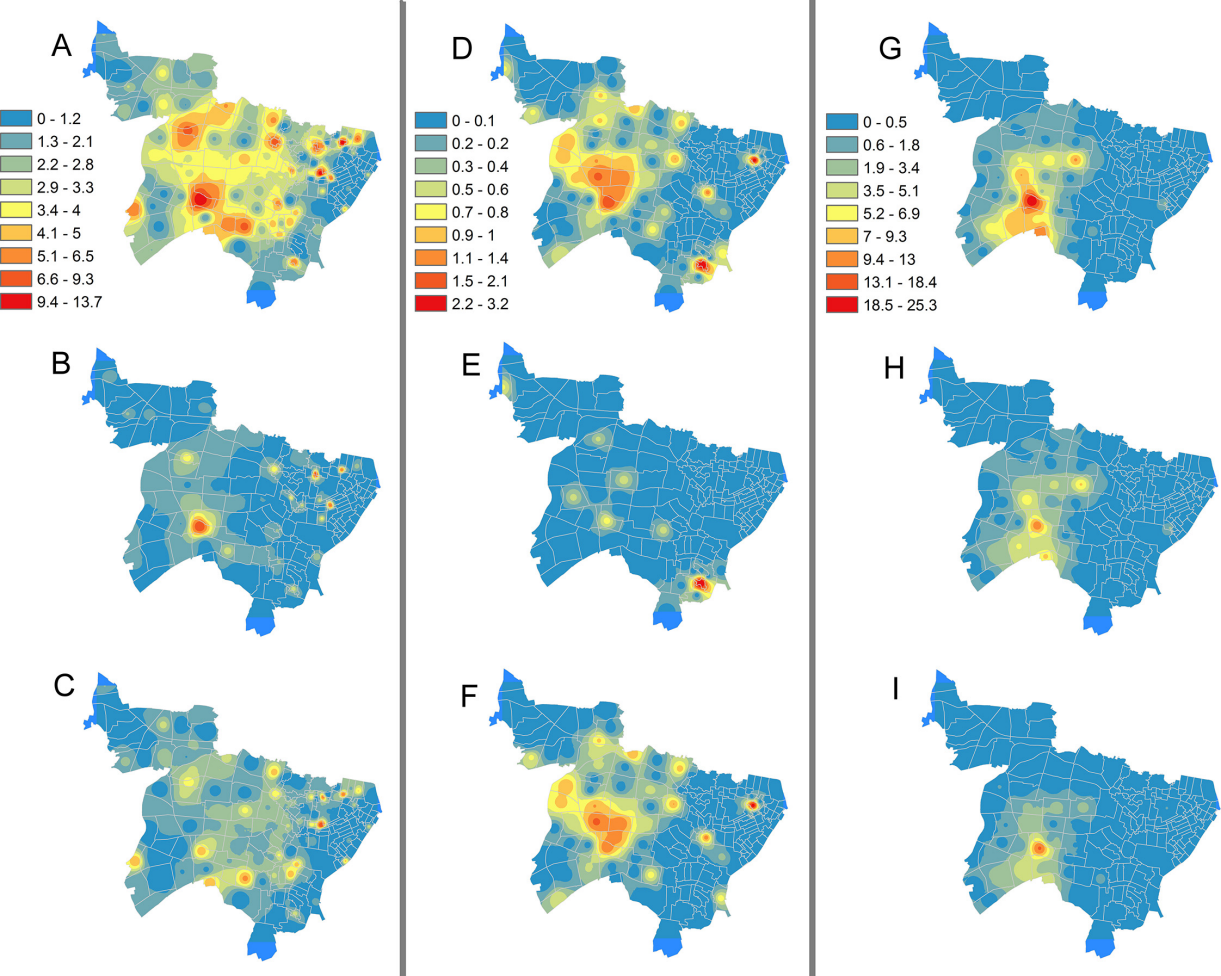
Inverse Distance Weighted interpolated maps with annual number of tuberculosis patients per square kilometer, stratified by residential status and genotype cluster status. Note: (A) General inhabitants. (B) Genotype-clustered general inhabitants. (C) Genotype non-clustered general inhabitants. (D) Foreign-born people. (E) Genotype-clustered foreign-born people. (F) Genotype non-clustered foreign-born people. (G) Homeless people. (H) Genotype-clustered homeless people. (I) Genotype non-clustered homeless people. Note: the color scale indicates the annual number of tuberculosis patients per square kilometer.

An IDW map identified a patient density peak (41.7/km^2^/year) near the Shinjuku railway station, which is one of the busiest railway transport hubs in Japan. Lower patient density areas were observed on the east and north sides of the city, both of which are residential zones (Fig 2). Various trends depended on different residential and genotype-cluster statuses were observed (Fig 3). Among the general inhabitants, a number of scattering peaks were found. The genotype-clustered group peaked slightly near the Shinjuku station, but few patients defused in the non-clustered group. Foreign-born people concentrated on the west side of the city where expat communities exist. However, as the number of patients was small, the peak (3.2/km^2^/year) was not as obvious as other residential groups. Homeless patients were aggregated around the station, regardless of genotype statuses.

### Hotspot Analysis

**Fig 4:**
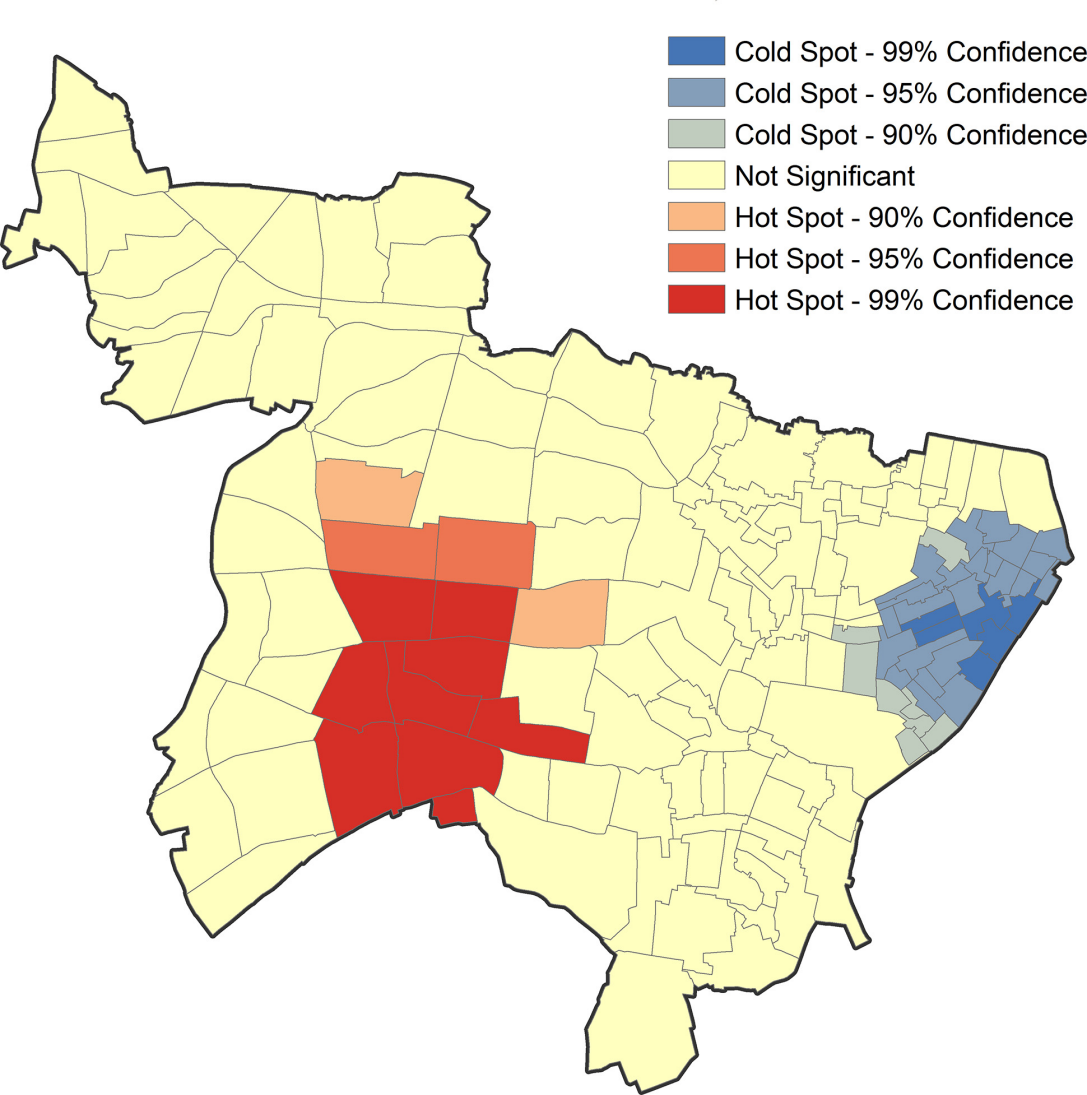
Hotspots and coldspots for all tuberculosis patients detected by Getis-Ords Gi^*^. Note: Three confidence levels (90%, 95%, and 99%) are provided; higher confidence levels imply stronger aggregation in either hotspots or coldspots.

The overall results showed that 13.7% of the study area (2.5 km^2^) was detected as hotspots and 6.3% as coldspots (1.1 km^2^) (Fig 4). Patients from hotspots tended to be male, <65 years old, homeless, unemployed, smear-positive, and genotype-clustered (Table 4). Compared to patients in outside of hotspots, they were diagnosed through contact investigations, welfare facility visits, or brought to the emergency department by ambulance.

**Table 4:** Comparison of characteristics of tuberculosis patients within hotspots and outside of hotspots of all study population including both genotype clustered and genotype non-clustered.

	Patients in hotspots, n = 240		Patients outside of hotspots, n = 403		*p*-value
Variables	n	(%)	n	(%)	
Male (excluded unknown = 11)	214	(89)	277	(70)	<0.01*
Age, years, mean (SD)	54	(16)	60	(20)	<0.01^†^
Age ≥65 years	63	(26)	194	(48)	<0.01*
Residential status					
General inhabitant	93	(39)	320	(79)	<0.01^‡^
Foreign-born people	16	(7)	26	(6)	
Homeless population	127	(53)	57	(14)	
Unknown	4	(2)	0	(0)	
Occupation					
Permanent worker	45	(19)	102	(25)	0.01^†^
Student	5	(2)	15	(4)	
Temporary worker	35	(15)	31	(8)	
Housewife	7	(3)	35	(9)	
Infant	0	(0)	1	(0)	
Unemployed	147	(61)	217	(54)	
Unknown	1	(0)	2	(0)	
Unemployed who were ≥65 years old	55	(23)	147	(36)	<0.01*
Smear-positive tuberculosis	162	(68)	217	(54)	<0.01*
Genotype-clustered	122	(51)	159	(39)	<0.01*
Diagnosis method					
Individual health check	3	(1)	15	(4)	<0.01^‡^
Periodic health check	13	(5)	42	(10)	
Contact investigation	11	(5)	0	(0)	
Other mass health check	3	(1)	9	(2)	
Health care facility visit	118	(49)	242	(60)	
Welfare facility visit, Emergency transportation, and others	73	(30)	35	(9)	
Health services other than TB^1^	14	(6)	49	(12)	
Unknown	5	(2)	11	(3)	

*Fisher's exact test

^†^Unpaired t test

^‡^Chi square test, SD = standard deviation

^1^Diagnosed as tuberculosis though inpatient or outpatient services for disease other than tuberculosis

We attempted to identify the urban environmental features of hotspots and coldspots, using nine variables (Table 5). The median of all population-related variables (except the percentage of 65-year-old and over residents) was higher in hotspots than in coldspots. In particular, hotspots were characterized by significantly higher daytime population density (*p* = 0.01) and density of foreign-born people (*p* = 0.02) than the coldspots. Households in hotspots had median 28.2 m^2^ floor area per capita, which was significantly smaller than household in coldspots (34.4 m^2^). The distance to the nearest railway station was shorter in hotspots (median = 359.8 m) than in coldspots (median = 549.4 m), but the difference was not statistically significant (*p* = 0.10). Percentage of non-working household (household in which no one held paying employment) was significantly higher in hotspots (56.9%) than in coldspots (35.1%).

**Table 5:** Comparison of urban environmental factors in hotspot and coldspot census tracts for all tuberculosis patients.

	Total CTs = 152						
			Hotspots^*^ CTs = 13		Coldspots CTs = 36		
Variables	median	IQR	median	IQR	median	IQR	*p*-value
**Population**							
Daytime population density (/km²)	27,675.0	(28,794.9)	63,589.5	(85,746.1)	31,200.0	(38,791.7)	0.01
Density of foreign population (/km²)	950.0	(1,344.1)	3,406.3	(5,870.2)	633.3	(958.3)	0.02
Day and night population ratio	149.0	(285.0)	445.0	(1,294.0)	195.0	(448.0)	0.07
Percentage of population aged 65 and over (%)	18.9	(5.0)	18.7	(4.9)	19.0	(11.3)	0.55
**Household Census**							
Household density (/km²)	10,352.2	(6,899.0)	10,128.6	(7,118.2)	8,300.0	(7,874.2)	0.73
Total floor area per capita (m²)	30.5	(6.0)	28.2	(3.9)	34.4	(5.9)	0.01
Percentage of owned house (%)	38.7	(15.6)	37.0	(13.7)	47.5	(28.2)	0.22
Percentage of non-working households (%)	40.0	(12.4)	56.9	(8.0)	35.1	(15.6)	<0.01
Distance to the nearest railway station from a CT centroid (m)	669.3	(539.2)	359.8	(267.2)	548.8	(349.4)	0.10

CTs = Census tracts, IQR = Inter-quartile range

^*^Hotspots and coldspots were detected by Getis-Ord G_i_^*^ statistics, which implies that detected hotspots have high patient density and are surrounded by other features with high patient density.

**Fig 5:**
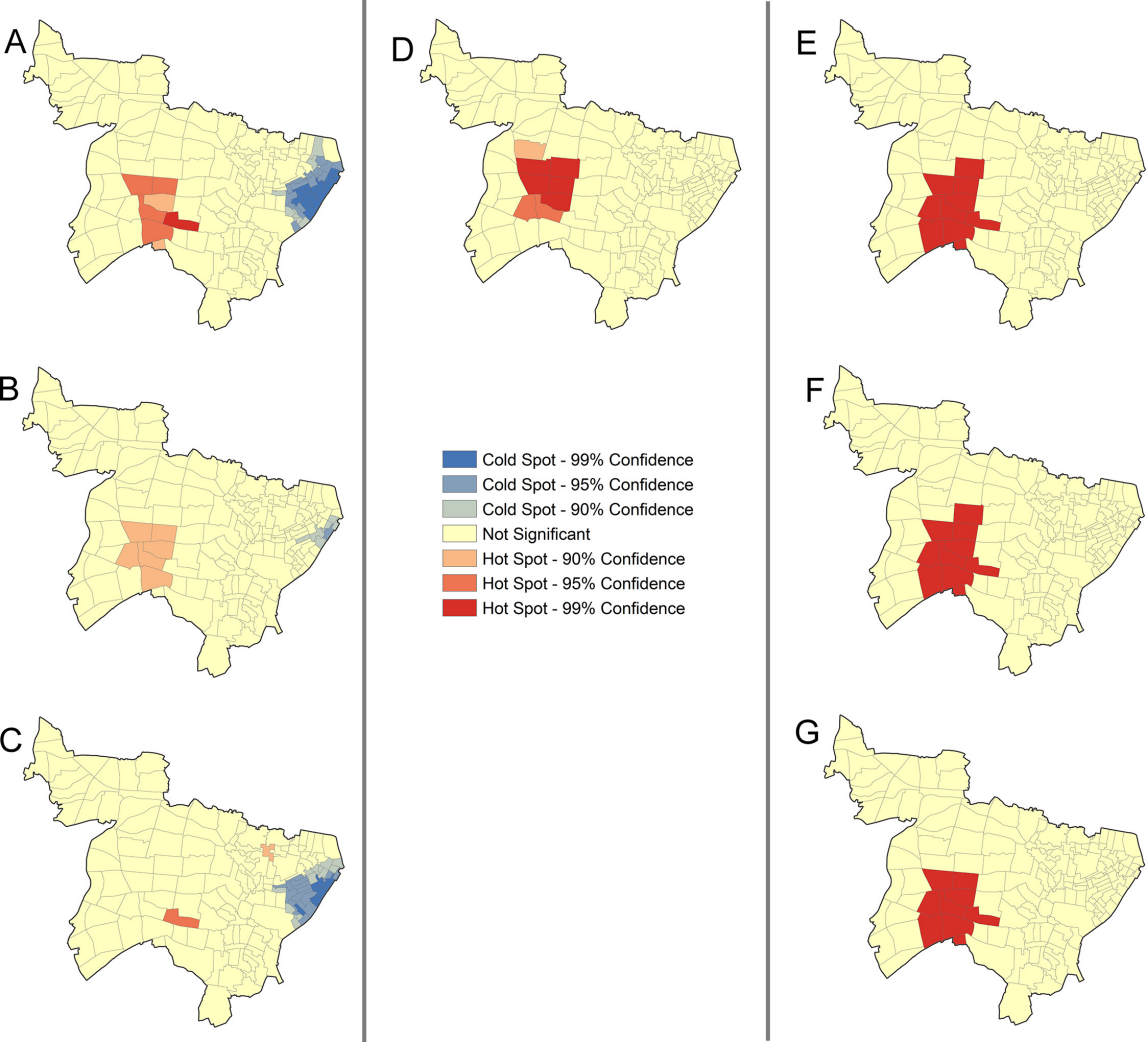
Hotspots and coldspots for tuberculosis patients detected by Getis-Ords Gi^*,^ stratified by residential statuses and genotype-cluster status. (A) General inhabitants. (B) Genotype clustered general inhabitants. (C) Genotype non-clustered general inhabitants. (D) Foreign-born people. (E) Homeless people. (F) Genotype clustered homeless people. (G) Genotype non-clustered homeless people. Note: Three confidence levels (90%, 95%, and 99%) are provided; higher confidence levels imply stronger aggregation in either hotspots or coldspots.

In the final part, Fig 5 showed hotspots and coldspots identified among different residential and genotype-cluster statuses. General inhabitants indicated similar patterns of hotspots and coldspots to the results of the overall group. Genotype-clustered general inhabitants formed a hotspot with a low confidence level (CI = 90%), whereas the non-clustered general inhabitants indicated almost no hotspots. Foreign-born people had obvious hotspots, but consisted of a few patients. We could not analyze by genotype-cluster status because of small sample size. Homeless people had significant hotspots around the station (CI = 99%), regardless of genotype cluster status.

## Discussion

We have hypothesized that transmission occurs in “activity space”, which may or may not be their residential homes. Our results showed that area surrounding Shinjuku station is significant hotspots for all patients regardless of genotype cluster and residential statuses. Similar area was identified as hotspots for both genotype clustered general inhabitants and homeless people, which indicate that recent transmission probably took place around Shinjuku station.

The identified hotspots could be characterized by distinct urban features, including large daytime populations, residence of a large proportion of foreign-born people, and proximity to the nearest railway station indicating large number of business and commercial facilities. At the same time, several indictors suggested low socioeconomic status of the hotspots, including smaller residence floor size, a higher proportion of non-working households, and, though not statistically significant, lower rate of homeownership.

Past studies in Japan have shown that TB transmission occur in public facilities and near downtown railroad station [21,22], and also that homeless and non-homeless people can share the same genotype cluster in an urban setting [4]. Patient interviews by the PHC staff have also revealed that many patients had used facilities such as saunas, bars, and pachinko halls, which have previously also been reported as TB outbreak venues [21]. The Tokyo Metropolitan Government published the Tuberculosis Control and Prevention Plan and pointed out that 89.2% of TB infections from 20’s to 50’s, while of unclear origin, were found in those who stayed a long time in the business facilities mentioned above [38]. In other high-incidence countries, epidemiological links between patients have been reported based on facilities used by unspecified numbers of people [15].

Area around Shinjuku station was also identified as a hotspot by the non-clustered homeless patients–this can partially be explained by the fact that Shinjuku city is one of the largest homes to homeless people, attracting people living under fragile conditions from around the country. In other words, some homeless people might have been infected prior to coming to Shinjuku, and hence do not belong to the same genotype cluster, but share similar activity spaces with the clustered homeless people, once they arrive in Shinjuku.

Lastly, the non-clustered general inhabitants were spread widely over the city, indicating no hotspots, as shown in Fig 3C. These areas consist largely of residential zone where retired elderly nominated as their activity spaces. In our study, 57% of genotype non-clustered patients were 60 years old and higher. This implies that TB developed from past (latent) infections and ongoing transmission of TB in the area was unlikely.

There are several limitations to our study. Firstly, we could only collect information regarding activity space close to the time of diagnosis, and not around the time of infection. Hotspot may, therefore, not necessarily represent a site of transmission. In order to partially overcome this issue, we conducted the analysis by genotype cluster statuses. Secondly, by utilizing the concept of one activity space per patient, we are assuming that TB transmission is most likely to occur in a place where the patient spends “most of his or her waking hours”. That is to say, our study does not take into consideration the possibility of transmissions occurring through casual contacts with the TB patient with minimal duration of contact, such as in a crowded elevator, which has been indicated as risk of transmission[39], and in public transport. Thirdly, the study population only included those who were registered with the Shinjuku PHC–however, considering the city’s large daytime population, we have potentially missed a large number of TB patients whose activity space is located in Shinjuku city but who reside elsewhere. A further research is therefore necessary to incorporate larger area, including the Greater Tokyo Area. Lastly, discussion of risk of TB transmission, such as incidence rate, in the hotspots was out of scope of the study, because appropriate population at risk based on activity spaces was not available.

Despite these limitations, our study used a novel concept of activity space to attempt to identify possible places of TB transmission, which could take into account both inside and outside household. Molecular epidemiological techniques have certainly made much contribution to understanding the dynamics of TB transmission, however, it is also true that epidemiological links are uncertain among some of the cases even identified as same genotype clustered group[3]. We have shown that activity space-based spatial analysis provided an alternative approach to residential address-based approaches, and that it can be used in combination with molecular technique in further comprehension of TB transmission dynamics in an urban setting in Japan.

## Conclusion

This study indicated possible sites of TB transmission in inner metropolitan setting, Shinjuku city, Tokyo, based on activity spaces using spatial analysis combined with molecular technique, which were characterized by relatively lower some socioeconomic factors. The most difficult but crucial question for breaking the chain of transmission is where ongoing TB infection occurs. Despite accompanied by several methodological limitations, activity space-based spatial analysis provided a further insight of TB transmission dynamics in an urban setting in Japan.
